# Can the material properties of regenerate bone be predicted with non-invasive methods of assessment? Exploring the correlation between dual X-ray absorptiometry and compression testing to failure in an animal model of distraction osteogenesis

**DOI:** 10.1007/s11751-014-0188-9

**Published:** 2014-03-05

**Authors:** Fergal Monsell, Andrew William Hughes, James Turner, Michael C. Bellemore, Lynne Bilston

**Affiliations:** 1 Bristol Royal Hospital for Children, Paul O’Gorman Building, Upper Maudlin Street, Bristol, BS2 8BJ UK; 2 Bristol Royal Infirmary, Bristol, UK; 3 Royal Alexandra Hospital for Children, Sydney, NSW Australia; 4 Neuroscience Research Australia, University of New South Wales, Randwick, NSW Australia

**Keywords:** Distraction osteogenesis, DXA, Bone mineral density, Animal model

## Abstract

Evaluation of the material properties of regenerate bone is of fundamental importance to a successful outcome following distraction osteogenesis using an external fixator. Plain radiographs are in widespread use for assessment of alignment and the distraction gap but are unable to detect bone formation in the early stages of distraction osteogenesis and do not quantify accurately the structural properties of the regenerate. Dual X-ray absorptiometry (DXA) is a widely available non-invasive imaging modality that, unlike X-ray, can be used to measure bone mineral content (BMC) and density quantitatively. In order to be useful as a clinical investigation; however, the structural two-dimensional geometry and density distributions assessed by DXA should reflect material properties such as modulus and also predict the structural mechanical properties of the regenerate bone formed. We explored the hypothesis that there is a relationship between DXA assessment of regenerate bone and structural mechanical properties in an animal model of distraction osteogenesis. Distraction osteogenesis was carried out on the tibial diaphysis of 41 male, 12 week old, New Zealand white rabbits as part of a larger study. Distraction started after a latent period of 24 h at a rate of 0.375 mm every 12 h and continued for 10-days, achieving average lengthening of 7.1 mm. Following an 18-day period of consolidation, the regenerate bone was subject to bone density measurements using a total body dual-energy X-ray densitometer. This produced measurement of BMC, bone mineral density (BMD) and volumetric bone mineral density (vBMD). The tibiae were then disarticulated and cleaned of soft tissue before loading in compression to failure using an Instron mechanical testing machine (Instron Corporation, Massachusetts USA). Using Spearman rank correlation and linear regression, there was a significant correlation between vBMD and the Modulus of Elasticity, Yield Stress and Failure Stress of the bone. No correlation was seen between BMC, BMD, vBMR and any mechanical parameter. DXA is a promising tool for the assessment of regenerate bone formed by DO during limb lengthening and requires further investigation.

## Introduction

 Distraction osteogenesis (DO) is a process of generating bone and is used to lengthen limbs and reconstruct skeletal deformities using techniques codified by Ilizarov [1–4]. This involves a process of bone division and gradual distraction, usually with an external fixator, to create “regenerate” bone. This is followed by a period of consolidation to allow the regenerate bone to become structurally competent and therefore tolerate axial load in weight bearing. Premature removal of the external fixator is associated with a risk of fracture or deformation of the regenerate bone with a reported incidence of between 3.6 and 24 % [5–8].

A number of testing configurations including tension, compression, torsion and three-point bending have been used to evaluate the biomechanics of bone in vitro. In a clinical case series following distraction osteogenesis, it was noted that the early fractures were associated with compression and partial collapse of regenerate [6]. This is distinct to diaphyseal bone that tends to fail in torsion. In this study, we used axial compression as it is a reproducible method of testing mechanical parameters and is clinically applicable to the mode of failure of regenerate bone.

An accurate, non-invasive method of assessment of the structural properties of the regenerate bone is essential to determine the appropriate time for removal of the fixator. Several methods have been described and include: plain radiography, digital radiography, quantitative computed tomography (QCT), dual-energy X-ray absorptiometry (DXA) and ultrasound (US) [8–12]. There is no single method of assessment that is in widespread clinical use.

DXA is a non-invasive method of assessing bone mineral density that is readily available in clinical practice. Over the last two decades, its use has increased dramatically, specifically in the assessment of osteoporosis in the ageing population. Eyres et al. [13] first described the use of DXA to quantify and monitor regenerate bone formation during leg lengthening and detected regenerate bone within 2 weeks of distraction, in contrast, regenerate bone was not detected on plain radiographs until 6 weeks.

The aim of this study is to investigate the correlation between the material characteristics of regenerate bone produced in a rabbit model of DO, as measured in a non-invasive manner with DXA, with the mechanical parameters measured by axial loading to failure. The clinical relevance is to determine whether it is possible to make an indirect assessment of the structural integrity of the bone using a readily available, non-invasive imaging technique.

## Material and methods

Lengthening of the tibia was performed in 41 male juvenile New Zealand white rabbits as part of a larger study investigating the effect of cyclical chemotherapy on DO [14].

All animals had a mid-diaphyseal tibial osteotomy and application of Orthofix M100 mono-lateral fixator (Orthofix, Bussolengo, Italy). Animals were sacrificed at 16 weeks of age, representing approximately 75 % completion of growth and simulating the effect of DO on a human adolescent [15]. The tibia was distracted at the rate of 0.375 mm every 12 h and distraction continued for 10 days, aiming for an overall lengthening of 7.5 mm. Consolidation of the regenerate bone occurred over a period of 18-days following distraction.

Bone density measurements were made using a total body dual-energy X-ray densitometer (LUNAR DPX, LUNAR Radiation Corp., Madison, WI) with software designed for measuring small animals (LUNAR DPX, Small Animal Software version 1.0, LUNAR Radiation Corp., Madison, WI). This machine used a constant potential X-ray source (76 kV) and a K-edge filter (cerium) to produce stable dual-energy X-rays with effective energies of 38 and 70 keV. A scan speed setting of “HiRes <0.5 kg Slow” was used, with a sample size of 0.6 × 1.2 mm, sample interval of 1/16 s, and an X-ray beam collimation of 0.84 mm at the source.

**Fig. 1:**
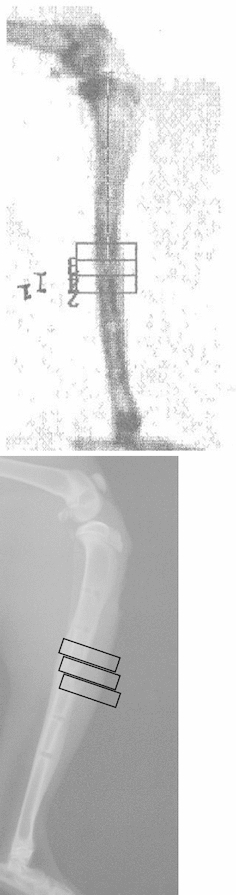
DXA image with corresponding radiograph

The disarticulated right hind limb was assessed with all soft tissues intact. The scan length was determined by the length of the bone (the scan area was approximately 50 mm × 130 mm). Each leg was placed in a supine position on the scan table, and a cranio-caudal (anterior-posterior) scan was performed. The leg was then medially rotated and a lateral scan was performed. Bone mineral density (BMD), bone mineral content (BMC) and bone area (BA) values were obtained with the “manual analysis” facility. The height of the boxes was determined by the height of the regenerate, and all box widths were identical (15 mm). Using the software “ruler”, the distance from the knee to the top of the regenerate and the height of the regenerate were measured and recorded. A “region of interest” (ROI) box was placed over the regenerate. The height of the ROI was such that the entire length of the regenerate was included. The boxes were positioned so that all the bone and some soft tissue were included in the region of interest (Fig. 1).

The software calculated BMC (g), BMD (g/cm^2^), average bone width (cm) and average bone area (cm^2^). vBMD was calculated manually assuming that the bone was an elliptical cylinder.

$$\text{The area of the cross - section of bone}=\Pi \times \frac{\text{AP Bone Width}}{2}\times \frac{\text{Lat Bone Width}}{2}$$

The volume of the region *V* = Cross-sectional Bone Area × ROI HeightThis assumes that the regenerate bone is homogenous throughout the ROI. The plain radiographs suggest that this is a reasonable assumption.

$$\text{Volumetric BMD(g}/{\text{cm}}^{3})=\frac{\text{BMC}}{V}$$

Each scan produced the following data from the AP and Lat scans

- Bone width (mm)
- Bone area (mm^2^)
- Bone mineral content (g)
- Bone mineral density (g/mm^2^)
- Volumetric bone mineral density (g/mm^3^)

From this data, the following were calculated:

$$\text{BMC}\frac{\left(\text{Lat BMC}+\text{AP BMC}\right)}{2}$$

$$\text{BMD}\frac{\left(\text{Lat BMD}+\text{AP BMD}\right)}{2}$$

$$\text{vBMD}\frac{\left(\text{Lat vBMD}+\text{AP vBMD}\right)}{2}$$

**Fig. 2:**
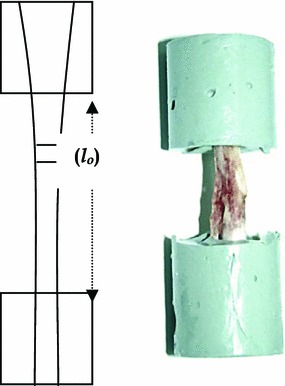
Mechanical test specimen and geometry

Prior to the mechanical testing, the ankle and knee were disarticulated and all soft tissues were removed, leaving the tibio-fibular complex. This was embedded in Permatex resin (Fig. 2) (Permatec inc, Hertford, Connecticut, USA) in a mounting block, then placed in an Instron mechanical testing machine (Instron Corporation, Massachusetts USA) and loaded in compression to failure at a rate of 2 mm/min using a 10-kN load cell.

This measured displacement (mm) for increasing load (Newtons) until failure, and the data were saved as an Excel spreadsheet and exported to Easy Plot (Spiral Software Norwich, Vermont USA) and produced load displacement curves for each specimen.

The length of exposed bone (lo) was measured prior to compression testing (Fig. 2). The mean area of the regenerate was calculated from DXA measurements and the load/displacement data were transformed to produce a stress/strain graph from which range, mean and standard deviation were calculated for the following parameters (Table 1):

**Table:** 

Modulus of elasticity	*E* (GPa)
Energy at yield	*e*_Y_ (KJ)
Yield stress	*σ*_y_ (MPa)
Yield strain	*ε*_y_
Energy at failure	*e*_F_ (KJ)
Failure stress	*σ*_f_ (MPa)
Failure strain	*ε*_f_

## Results

DXA parameters (BMC, BMD and vBMD) were compared with mechanical parameters (*E*, *e*_Y_,*σ*_y_, *ε*_y_, *e*_F_, *σ*_f_, *ε*_f_) for all specimens using Spearman rank correlation and simple linear regression. All rabbits were males and were 12 weeks old at the time of osteotomy. The average lengthening was 0.71 cm (SD 0.12), giving a regenerate area of 0.61 cm^2^ (SD 0.21) and volume 0.44 cm^3^ (SD 0.20). There was no significant correlation between BMC, BMD and any mechanical parameter (Table 2).

**Table 1:** Mean and SD of all mechanical and DXA parameters

	Mean	SD
BMC (g)	0.28	0.11
BMD(g/cm^2^)	0.43	0.08
v BMD(g/cm^3^)	0.63	0.09
Young’s E (GPa)	0.55	0.235
eY (kJ)	30.7	13
Y stress (MPa)	17.1	4.55
Y strain (mm/mm)	0.04	0.01
eF (kJ)	63.6	52.7
F stress(MPa)	19.2	4.5
F strain (mm/mm)	0.05	0.02
Post-yield e (kJ)	32.8	52.6

**Fig. 3:**
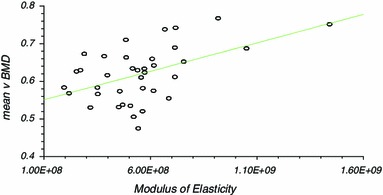
Linear regression analysis for modulus of elasticity

**Fig. 4:**
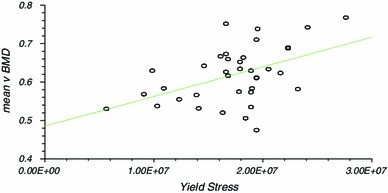
Linear regression analysis for yield stress

**Fig. 5:**
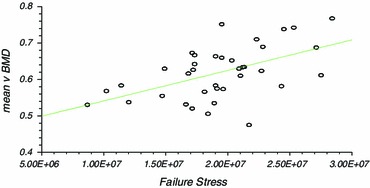
Linear regression analysis for failure stress

There was significant correlation between vBMD and modulus of elasticity (Correlation coefficient *r* = 0.50, *r*^2^ = 0.25, CI 0.21–0.71, *P* ≤ 0.01), yield stress (*r* = 0.47, *r*^2^ = 0.22, CI 0.16 to 0.69, *P* ≤ 0.01) and failure stress (*r* = 0.53, *r*^2^ = 0.26, CI 0.24 to 0.73, *P* ≤ 0.01). The results of linear regression analysis are presented (Table 2; Figs. 3, 4, 5) as they were considered to be more useful in that an estimation of the mechanical characteristics could be calculated by interpolation.

**Table 2:** Linear regression analysis

	Correlation coefficient (r)	95 % CI for r (Fisher’s z transformed)	Two sided *P*	Power (for 5 % significance) (%)
*v*BMD versus modulus of elasticity	0.50 (*r*^2^ = 0.25)	0.21–0.71	<0.01	88.23
*v*BMD versus yield stress	0.473 (*r*^2^ = 0.223)	0.16–0.69	<0.01	84.14
*v*BMD versus failure stress	0.53 (*r*^2^ = 0.26)	0.24–0.73	<0.01	92.02

## Discussion

This is the first animal study to demonstrate a statistically significant correlation between DXA and mechanical parameters for regenerate bone determined by compression testing. The correlation between this non-invasive investigation of regenerate material properties and mechanical properties may have clinical implications. Failure under axial load is the most common mode of regenerate bone failure and fundamentally differs from fracture in normal bone. A non-invasive method of estimating this mechanical property could therefore identify incompetent regenerate and potentially reduce complications related to premature external fixator removal.

Plain radiography has been used to evaluate the regenerate bone during limb lengthening, bone transport and deformity correction. It is useful in overall assessment of bone alignment, length and width of regenerate bone but cannot detect the potential complications of failure of early distraction gap consolidation or delay in regenerate bone formation [16]. With the advent of digital radiography, it has been postulated that it is possible to quantify the mechanical properties of the regenerate. Kolbeck et al. [9] reported a high correlation between digital radiographic densimetric evaluation of regenerate and torsional force measurements in a micropig animal model of distraction. Shim et al. [8, 17] have assessed digital radiography and picture archiving and communication systems (PACS) in clinical practice. They used relative pixel value ratios of regenerate bone to that of original cortex and found changes in bone density followed a sigmoid curve pattern. In their cohort of patients, the average healing index was 59 days/cm, more than 10 days/cm longer than previously published clinical research using DXA to evaluate regenerate [8]. The higher healing index is clinically significant as delayed external fixator removal can lead to osteoporosis and fractures unrelated to the regenerate, increased opportunity for pin site infection and an adverse psychological and socio-economic impact to the patient.

DXA is a non-invasive semi-quantitative method of measuring bone mineral content and aspects of density. It has been proposed as a low-exposure radiological investigation that could be used to assess rate of new bone formation and strength of regenerate bone in DO. Eyres et al. [13] first described the use of DXA to quantify and monitor regenerate bone formation during leg lengthening and detected regenerate bone formation within 2 weeks of distraction, in contrast, regenerate bone was not detected on plain radiographs until 6 weeks. There was a linear increase in bone density over the first 3 months after maximal distraction, with progressive slowing towards final consolidation. They also demonstrated that DXA could not always detect defects in the regenerate and concluded that ultrasound was excellent at detecting these defects and therefore complemented DXA during limb lengthening, reducing the need for plain radiography [10, 13, 18]. Saran et al. reported that the use of monthly DXA scans during the consolidation phase of limb lengthening has a low rate (3.6 %) of fractures following frame removal, maintaining an acceptable bone healing index without excessively increasing fixation time. Fixators were removed when bone mineral density had plateaued to less than 10 % increase and plain radiographs showed no obvious defects precluding fixator removal [8]. Paley advised frame removal when three of four cortices are seen in the regenerate bone column on plain radiographs or evidence of neocorticalisation and opacity similar to its surrounding bone are seen [19]. Saran however removed fixators on the basis of DXA alone with an average of 1.3 cortices formed at the time of removal and experienced no fractures in these patients [8].

This study showed a significant association between vBMD and the modulus of elasticity, yield stress and failure stress of regenerate bone in a rabbit DO model. We did not show a statistically significant correlation between BMC, BMD and the modulus of elasticity, yield stress and failure stress of the bone. Chotel et al. [11] compared bone stiffness, using a 3-point bending test with an orthometer, and DXA in children undergoing lengthening by DO. There was a poor correlation if values for DXA data were used as absolute values without correction for regenerate volume. When BMC was expressed as a percentage of the reference value, taken in a symmetrical region of the contralateral side, a linear correlation between BMC measurements and stiffness was identified. In our study, we did not use the contralateral limb as a reference and this may explain the lack of correlation between BMC and BMD and mechanical parameters. Reichel et al. [20] studied DXA and mechanical parameters in an ovine model of DO using non-destructive axial compression testing and a torsional force to failure. They did not demonstrate a significant correlation between axial compression and BMD; however, there was a strong correlation between maximum torque and BMD.

In our institution, DXA is readily available in clinical practice and using small animal software, provided a simple and reproducible method of analysis. Alternative methods of assessment include pQCT (peripheral Quantified Computerised Tomography) in vivo and micro-CT post-mortem. These methods provide information on the spatial distribution of bone and allow more sophisticated analysis of the structure of the regenerate. pQCT is becoming more accessible and may provide a superior and more straightforward method of assessment. It is possible that failure of regenerate occurred through a fibrous inter-zone in this animal model, which may not necessarily be detected by DXA and plain radiography. Micro-CT would have overcome this and should form the basis of future work.

The principal advantage of pQCT and micro-CT over DXA relates to the resolution and accuracy of assessment of the three dimensional structure of bone [21]. pQCT has a field view of 5–15 cm with a resolution of 100–1,000 μm and allows assessment of peripheral sites in intact experimental animals and determination of BMD with assessment of trabecular architecture parameters. Micro-CT has a field of view between 1 and 5 cm with a resolution of 10–100 μm and allows assessment of complete trabecular architecture in small ex-planted samples or small animal peripheral sites in vivo. DXA does not account for the spatial distribution and inherent material properties of the tissues, and pQCT would have offered a potentially superior method of assessment and should be considered as a method of assessment in future work.

Markel et al. [22, 23] developed a canine model to compare pQCT, SPECT (Single Photon Emission Computed Tomography) and DXA as a method of assessment of the torsional properties of healing tibial osteotomies. SPECT had the strongest association with maximum torque and torsional stiffness and pQCT and SPECT with indentation stiffness, but these were not significantly different from DXA.

The decision to use axial compression to test mechanical parameters was based on the mode of failure of regenerate bone in clinical practice [6]. This is distinct from intact bone which seldom fails because of axial compression [24]. Previous studies have demonstrated a relationship between structural and material properties of bone with this type of compression testing and a simple uniaxial loading test provided a reproducible method of analysis for this experiment [25, 26].

There are several alternative methods for measuring mechanical properties of intact bone in vitro, including tension, torsion and three-point bending. There is currently no consensus on the optimal experimental design for testing regenerate bone. Bending tests involve complex internal stress fields and produce results that are influenced by the testing skill and orientation of the specimen and 3- and 4-point bending tests present technical difficulties associated with sample positioning and the subsequent data analysis with irregular samples such as these. Testing would therefore be difficult to standardise and the results difficult to interpret. Furthermore, this type of testing, when applied to fractures or experimental osteotomies, has a tendency to combine the properties of the bone with those of the developing callus in an unpredictable manner also making analysis of the regenerate bone difficult.

Tensile testing has been shown to permit direct intrinsic determinations of tissue quality, but it was recognised by Black et al. [27] that this method of testing was associated with severe limitations, particularly with difficulties between bone clamp interfaces. Torque has been used to measure regenerate strength in various animal models. In one study by Pilla et al. [28], they commented on the limitations of torsional analysis, in particular the effect of increased surface area of the callus in osteotomised animals on the stiffness of the osteotomised specimens, which was greater than intact bone. Floerkemeir et al. [29] have established measurements of torsional, bending and compressive stiffness to be suitable as predictors of the load-bearing capacity of healing callus in animal models.

This study used uniaxial compression to failure, therefore testing the bone beyond the linear elastic range. There was a low correlation coefficient and coefficient of regression with wide confidence intervals; this reduces the significance of this correlation and thus reduces the applicability of these results to clinical practice. DXA measurement was confined to a single assessment for each tibia, and therefore, inter- and intra-rater reproducibility was not assessed introducing a potential bias.

With the varied options available to monitor regenerate bone formed by DO, a prudent approach in clinical practice would involve using more than one method. A balance needs to be reached between patient risk, cost, availability and reliability. Plain radiography is useful in assessing bony alignment following distraction, but even with newer digital radiography, it is still unable to accurately quantify the regenerate. DXA is a non-invasive procedure requiring minimal radiation that can be used alongside plain radiography to quantify the regenerate. This research did not study the changes in DXA measurements during early distraction, and therefore, we cannot comment on the value of DXA in the early distraction period. DXA is a promising tool for the assessment of regenerate bone formed by DO during limb lengthening and requires further investigation.
